# Potentiality of Utilizing Woven Pineapple Leaf Fibre for Polymer Composites

**DOI:** 10.3390/polym14132744

**Published:** 2022-07-05

**Authors:** Agung Efriyo Hadi, Januar Parlaungan Siregar, Tezara Cionita, Mohd Bakeri Norlaila, Muhammad Amin Mohd Badari, Agustinus Purna Irawan, Jamiluddin Jaafar, Teuku Rihayat, Ramli Junid, Deni Fajar Fitriyana

**Affiliations:** 1 Mechanical Engineering Department, Faculty of Engineering, Universitas Malahayati, Jl. Pramuka No. 27, Kemiling, Bandar Lampung 35153, Indonesia; 2 College of Engineering, Universiti Malaysia Pahang, Gambang 26300, Pahang, Malaysia; norlailamohdbakeri@gmail.com (M.B.N.); aminbadari3916@gmail.com (M.A.M.B.); ramli@ump.edu.my (R.J.); 3 Faculty of Engineering and Quantity Surveying, INTI International University, Nilai 71800, Negeri Sembilan, Malaysia; 4 Faculty of Engineering, Universitas Tarumanagara, Jakarta Barat 11440, Indonesia; agustinus@untar.ac.id; 5 Faculty of Mechanical and Manufacturing Engineering, Universiti Tun Hussein Onn Malaysia, Parit Raja, Batu Pahat 86400, Johor, Malaysia; jamiluddin@uthm.edu.my; 6 Department of Chemical Engineering, Politeknik Negeri Lhokseumawe, Lhokseumawe 24301, Indonesia; teukurihayat@yahoo.com; 7 Department of Mechanical Engineering, Faculty of Engineering, Universitas Negeri Semarang, Kampus Sekaran, Semarang 50229, Indonesia; deniifa89@mail.unnes.ac.i

**Keywords:** pineapple leaf, natural fibre, woven, layering number, fibre orientation

## Abstract

Pineapple leaf fibre (PALF) is one of the natural fibres with the highest tensile strength and cellulose content. This has led to the investigation of the application of short, long, random mats, and unidirectional types as reinforcement agents, but there is limited study on the usage of woven PALF in composites. Therefore, this study aims to investigate the potential of this woven PALF in reinforcing epoxy resin (ER) composite as well as the effect of layering numbers and fibre orientations on the mechanical properties of the product. This involved using hand lay-up and press moulding with a hydraulic machine to produce the composite and specimen test while 2, 3, and 4 layers of woven PALF were used as the layering number parameter. Moreover, the warp and weft direction of the woven PALF were used to simulate the effect of fibre orientation in composites. The findings showed that the 3-layer woven PALF performed better in terms of tensile and flexural properties than the other layers. It was also discovered that the orientation of the composite with warp direction is slightly higher than the weft direction. Furthermore, the scanning electron microscopic (SEM) method was applied to analyse the tensile fracture of the composites and the results showed that the interfacial adhesion of the 3-layer woven PALF successfully transferred the load to the epoxy resin matrix due to its reinforcement role in composites.

## 1. Introduction

The production of different types of materials to meet demands while considering both the economic and ecological importance has become very critical in the material engineering field. This is necessary due to the negative effect of the manufacturing and development of such products on the environment [1]. Moreover, composite materials are currently being developed to achieve greater strength and this involves mixing two or more materials to create another material. The combination of this technique with green technology indicates the importance of using renewable sources in maintaining sustainability. It is important to note that a natural fibre reinforcement composite has several advantages to meet the needs of industry and these include its lower price, being environmentally friendly, its possible use as a replacement for synthetic fibres, lower density, and higher mechanical ability [2]. The application of natural fibre in producing composite materials can improve sustainability by reducing energy consumption due to its low density [3].

The qualities of a finished product are determined by elements such as the content, type, orientation, and moisture content of the fibre, which are considered to have a substantial impact on the processing of natural fibre-reinforced polymer composites (NFRPC). Several studies have published findings on the parameters determining the physical and mechanical properties of natural fibre woven composites. Previous studies have also investigated the factors influencing the mechanical performance of these composites and reported the strong effect of different factors such as the woven structure, layering sequences, fibre orientation and type of polymers used [4,5,6]. Therefore, this study is focused on comprehensively discussing the potential factors of composites affecting the mechanical properties of the woven composite based on a previous study.

Khan et al., (2019) defined tensile strength as the highest value of stress a material can withstand under tension before it breaks or fractures [7]. The tensile properties of composites are affected by matrix damage due to loading and the interfacial adhesion of NFRPC [8]. This behaviour is related to better fibre adhesion in composites, which has led to the study of the comparison of a fracture surface under tensile load on two- and four-layer plain jute. The results showed that the matrix damage on the surface was due to the lower stress transfer from fibre to the matrix. It was concluded that the increasing number of layers of fibre in polymer composite increased material stiffness. This was further confirmed by previous studies such as Yahaya et al., (2015), who studied the composite of Kevlar/kenaf layering sequence and reported that four-layer Kevlar/kenaf had higher tensile strength and improved the tensile strength of the composites treated with woven kenaf compared to the three-layer sample [9].

The mechanical performance of hybrid woven NFRPC was investigated by Misnon et al., (2015) and Tezara et al., (2021) based on layering number and different orientations of fibre at 0°, 30°, 45°, and 90°. The findings showed that the fibre oriented at 0° exhibited the highest tensile and flexural strength compared to other orientations. It is, however, important to note that low tensile strength was observed to lead to poor fibre arrangement [10,11]. Another study by Yong et al., (2015) on the mechanical properties of woven ramie showed that the maximum tensile strength of woven ramie was influenced by warp (678 MPa) compared to weft (505.5 MPa). This means a good and efficient arrangement of fibre has the ability to provide stronger and higher tensile strength for the composite [12]. Moreover, the morphological and mechanical properties of woven natural fibre-reinforced thermoset polymer composites were studied by Malaiah et al., (2013) and Salman et al., (2015) with a focus on the effect of fibre type and orientation. The results showed that woven natural fibres and epoxy combination at 0°/90° fibre orientation had high flexural strength and modulus and this was possibly associated with the better compatibility of the fibres and epoxy matrix and their greater interface bonding [13,14]. Abdellaoui et al., (2015) also studied the mechanical properties of the laminated composite of jute and epoxy resin fabricated by compression using two fibre orientations at 0° and 45° and cutting directions at 0° and 45°. The mechanical tests were conducted on fabricated laminates at 1, 3, 5, and 7 layers and the results showed that the composites at 0° fibre orientation and cutting direction had higher mechanical properties [15].

This literature review indicates that fewer studies have been conducted on the properties of the woven pineapple leaf fibre (PALF) as well as on its mechanical performance when applied as a composite reinforcement. Furthermore, fewer integrated analyses were found on fibre stacking sequence woven PALF composites. These are the major research gaps in studying natural fibre composites and are considered to be challenging for future studies. Therefore, this study aims to analyse the mechanical properties of the woven pineapple leaf fibre based on the effect of layering number at two, three, and four-layer and fibre orientations of 0° and 90°. The focus on this natural fibre is due to its versatility and massive potential.

## 2. Experimental Method

### 2.1. Materials

The plain type of woven PALF used to fabricate the composite specimen and as reinforcement in epoxy resin was supplied by Pengrajin Fibre in Pemalang, West Java, Indonesia. The polymer matrix (epoxy resin) Epikote 816 and hardener 651A were purchased from Impiana Z Enterprise in Kuala Lumpur, Malaysia. Both products were manufactured by Hexion, United States.

### 2.2. Fabrication of Laminate Composite

**Figure 1:**
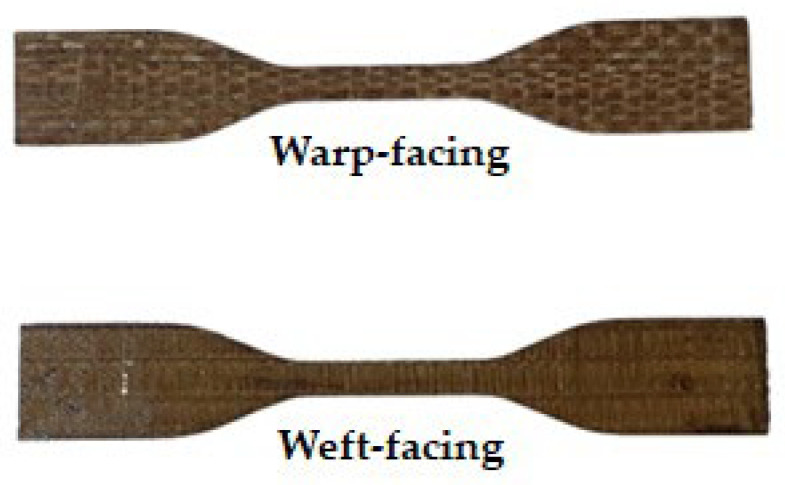
Warp and weft direction of woven PALF-reinforced epoxy composites.

A hand layup technique was used to fabricate the composite of three different layer numbers with woven PALF-reinforced epoxy composite. The process involved cutting the woven PALF into 300 × 300 mm and layering it up one layer at a time in aluminium moulds. A releasing agent was sprayed on the surfaces of the inner moulds before the lamination process was initiated in order to ensure the composite was not stacked to the moulds and to produce a smooth surface for the sample. Epoxy resin and hardener matrix mixed at a ratio of 3:1 was stirred slowly and uniformly by hand using a wooden stick for 5 minutes. The next step was to pour the epoxy resin into the mould and use the scraper to spread it out to the fibres’ surface, followed by the 2-layer, 3-layer, and 4-layer woven PALF. This was followed by the application of fifty bars of compression moulding machine to remove the air bubbles. The composites were cured for 24 hours at room temperature by applying compression pressure with dead weights on top of the mould. The samples were later retrieved from the mould and cut into the required size using a laser cutter according to the ASTM standard. The weights of the woven fibre, hardener, and epoxy used in the preparation process are tabulated in Table 1. Figure 1 illustrates the warp and weft orientation of woven PALF-reinforced epoxy composites.

### 2.3. Mechanical Testing

The samples used for the tensile and flexural tests were cut using a laser cutter machine. Those for the tensile test were prepared and machined according to the ASTM D638 Type IV procedure (ASTM, 2014). The dog bone-sided tensile samples fabricated were cut into an overall length of 115 mm and a thickness of 3 mm and placed on an INSTRON 3369 universal testing machine (UTM) with a crosshead speed of 2 mm/min and a load of 50 KN. Meanwhile, the flexural test specimens were prepared according to the ASTM D790 with the flexural strength and flexural modulus estimated by performing rectangular-shaped three-point bending tests on a sample with a dimension of 127 mm × 12.7 mm × 3 mm using an INSTRON 3369 universal testing machine with a 2 mm/min crosshead speed at room temperature.

### 2.4. Scanning Electron Microscopy (SEM)

The Zeiss Evo50 scanning electron microscope was utilised to examine the fibre surface and fracture morphological behaviour of tensile fracture specimens in woven PALF-reinforced epoxy composites. The samples were sputter-coated with a thin coat of palladium and placed on an SEM holder using double-sided electrically conductive carbon adhesive tapes to avoid surface charge when exposed to the electron beam. Finally, a microscope with a magnification of ×180, ×300 and a 15 kV acceleration tension was used to analyse the samples.

## 3. Result and Discussion

### 3.1. Tensile Test

The average tensile properties such as tensile strength (TS) and tensile modulus (TM) of the woven PALF-reinforced epoxy composite were investigated at different layering numbers, i.e., 2-layer (2L), 3-layer (3L), and 4-layer (4L), and two different fibre orientations of warp (0°) and weft (90°). The results were recorded and summarized for analysis and evaluation. It is important to note that standard deviations were also included in the graphs.

**Figure 2:**
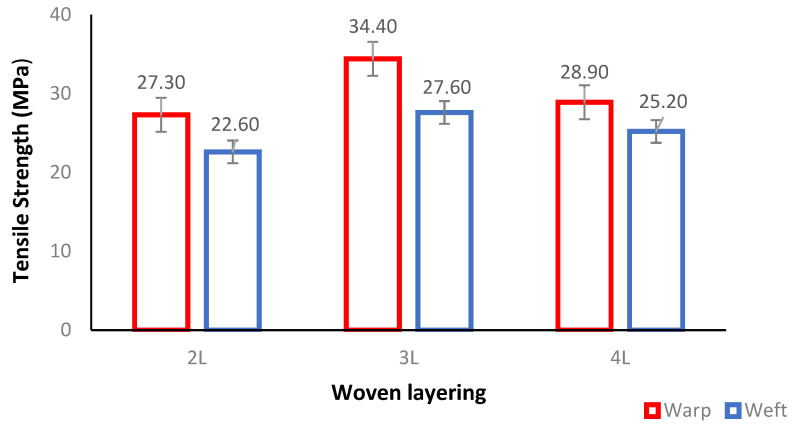
Tensile strength of woven PALF-reinforced epoxy composite at different layering numbers and fibre orientations.

Figure 2 shows that the woven PALF-reinforced epoxy composite specimens with warp fibre orientation have the highest tensile strength while those with weft orientation had the lowest average. This is due to the good and efficient arrangement of fibre which offers the composite a stronger and higher tensile strength [16]. This is in line with the findings of Tezara et al., (2021), who found that the fibre oriented in the warp direction exhibited the highest value of tensile strength compared to other orientations using layering numbers at different orientations of 0°, 30°, 45°, and 90° [4].

It was discovered that the tensile strengths of 2L-warp and 3L-warp specimens were, respectively, 27.30 MPa and 34.40 MPa, while the tensile strengths of 2L-weft and 3L-weft were, respectively, 22.60 MPa and 27.60 MPa. This represents a reduction of 17.20% and 20% for 2L and 3L, respectively. The values for the fibre orientation increased by 20% points from 2L to 3L for the warp, and by 22% points for the weft. It is also important to note that the increasing number of layers for both orientations, probably as a result of zone stress, led to an increase in the composite materials and caused a decohesion between the fibre matrix under stress. This is something that should be noted [17]. This was observed to be similar to the observations of a previous study by Abdellaoui et al., (2015), which stated that an increase in the tensile strength linked to layering number composite of 0° and 45° jute fibre direction was due to an increase in the zone stress. This was observed to be similar to the observations of the previous study [15].

However, it was found that the tensile strength of the woven PALF-reinforced epoxy composite decreased when the woven fibre exceeded the critical limit of 3L. This was related to the inability of the composite materials to hold the reinforcement of the polymer matrix beyond the critical fibre composition, which has a minimal stress transfer between the fibre and matrix. These results were found to be consistent with the findings of a previous study that compared the mechanical properties of composite kenaf and PALF. In that study, it was found that the mechanical strength of kenaf-based composites decreased when the fibre exceeded a critical value for tensile strength, which resulted in higher mechanical strength for the composites. [18]. In this study, the tensile strength dropped from 3L to 4L by 16% for warp fibre orientation and by 9% for weft fibre orientation. The poor arrangement of the fibres in 4L is probably to blame for its lower tensile strength.

**Figure 3:**
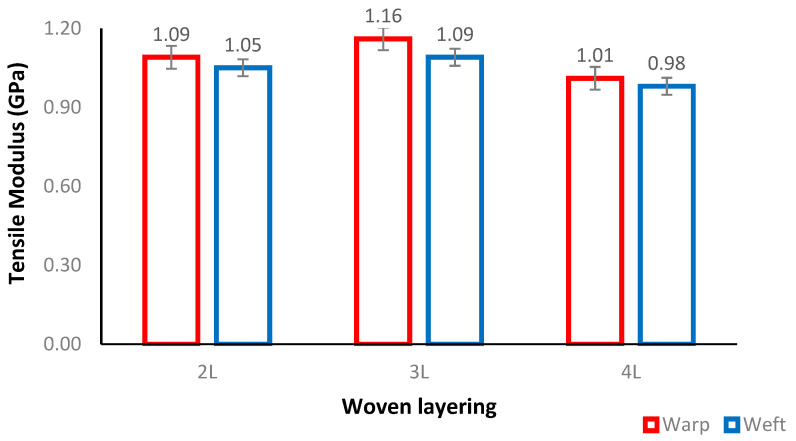
Tensile modulus of woven PALF-reinforced epoxy composite at different layering number and fibre orientations.

Figure 3 demonstrates that the average tensile modulus of woven PALF-reinforced epoxy composites follows the same trend as the tensile strength. As the number of woven layers increased from 2L to 3L, the average tensile modulus value for both the warp and weft fibre orientations increased. The maximum 4L woven PLAF exhibited a lower modulus of tensile strength. This decrease is likely due to the incorporation of defects during the layered fabrication process, such as pores and inadequate interface fiber–matrix bonding. The specimen with warp fibre orientation has the highest value, which is consistent with the findings of Tezara et al. [4]. Table 2 summarises the results of previous research on the tensile properties of fibre-reinforced epoxy composites with a variety of layering numbers and natural fibre types.

The 2L layering number had a 1.09 GPa tensile modulus for the warp and 1.05 GPa for the weft orientation and these were 6% and 17.36% lower than the values obtained by 3L. This was supported by the findings of Abdellaoui et al., (2015), where a higher tensile modulus was reached at five layers of woven jute fibre-reinforced epoxy composites due to the perfect adhesion between the fibre and matrix [15]. Meanwhile, 4L has the slightly lowest tensile modulus with 0.89 GPa for warp and 0.88 GPa for weft. This means the 3L composite is more ductile than the 4L. Therefore, it can be concluded that the 3L woven PALF epoxy composite performed very well compared to other layering numbers in the two fibre orientations.

Previous research revealed that the maximum tensile strength achieved ranges from 27.30 MPa for 2 layering numbers to 124.72 MPa for 6 layering numbers, while the tensile modulus ranges from 1.01 GPa to 2.98 GPa. The data also revealed that the woven PALF-reinforced epoxy composite with 4 layering numbers in Karimzadeh et al., (2020) possessed superior tensile properties with 47.07 MPa for TS and 2.98 GPa for TM compared to the results of the present study, which had 28.90 MPa for TS and 0.93 for TM, indicating a difference of 39.69% and 70.13%, respectively [22]. The weaker tensile strength observed in 4L is likely due to the poor arrangement of its fibres. It was also discovered that improper matrix-to-fibre bonding led to a decrease in tensile properties due to the ductility of epoxy resin in withstanding greater loads before breaking [15]. This comparison, therefore, showed that the tensile properties of composites are affected by the woven fibres, weave designs, fibre orientations, and layering numbers.

### 3.2. Flexural Properties

**Figure 4:**
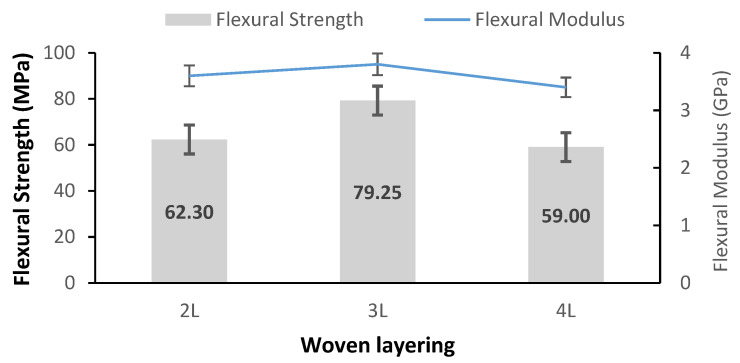
Flexural properties of woven PALF-reinforced epoxy composite at different layering numbers.

The flexural test was performed to measure the material’s ability to withstand bending up to the point of failure. Figure 4 illustrates the effect of the number of layers on the flexural strength (FS) and flexural modulus (FM) of the woven PALF-reinforced epoxy composites. It was discovered that the FS and FM increased when the number of woven layers increased from 2L to 3L. This was evidenced by the fact that the 3L specimens had the highest flexural strength of 79.25 MPa and flexural modulus of 3.8 GPa in comparison to the other layering numbers. The increases in FS and FM from 2L to 3L were 27% and 17%, respectively. This study’s result is consistent with the findings described by Rajesh and Pitchaimani [23]. The flexural strength will be improved as the content of woven layering is increased [24].

It was also observed that the value of flexural strength and flexural modulus decreased by 26% and 11% from 3L to 4L. According to the findings from the previous studies, a lowest flexural strength indicates that a minimal amount of stress was transmitted from the matrix to the fibres [25]. This is comparable to the findings of Rassiah et al., (2018) who discovered that the flexural strength and flexural modulus of woven bamboo *Gigantochloa Scortechiniithe* reinforced epoxy composite decreased with the increase of woven layering number from 2L to 6L. From their study, they concluded that the failure of bamboo fibre to support the stresses that had been transferred from the epoxy matrix likely caused the structure’s weakness. In addition, poor interfacial adhesion may have developed between the bamboo and the epoxy matrix, particularly as the bamboo layer became thicker [26]. This study revealed that the FS and FM of the woven PALF composite were affected by the number of layers and fibre–matrix adhesion strength in the samples. Flexural strength refers to the combination of tensile and compressive strength that varies directly with interlaminar shear strength, whereas flexural modulus measures the composites’ resistance to bending deformation [8,27].

Table 3 summarises the findings of past studies on flexural properties of fibre-reinforced epoxy composites at different layering numbers and a decreasing trend was observed for average flexural strength as the number of woven layers increased, as indicated: 6L (52.25 MPa) < 4L (78.04 MPa) < 3L (79.25 MPa). This means that the alteration of the woven fibre arrangement affected the flexural properties and also shows that an increase in the number of layers of reinforcement is very significant in studying flexural properties [19]. Meanwhile, the flexural modulus was discovered to increase with an increase in the layering number but dropped when it reached a critical limit of 6L due to the failure of the fibre matrix and delamination [6].

PALF fibre parameters such as fibre loading weight percent or volume fraction and fibre type have been found to have a significant influence on the mechanical properties of a variety of epoxy polymer-based composites. Table 4 shows that some conflicting results have been found in a few instances for the same parameter when a different combination of composites was used. These findings were based on observations. For instance, in the fibre loading parameter from Table 4, it was discovered that the highest tensile properties were obtained for the 30 wt.% fibre loading, whereas for other composite combinations, it was at 20 wt.% fibre loading. This was found to be the case for the material. This was because of the contribution of a number of different factors, such as type of the arrangement of the fibres and the length of the fibres.

### 3.3. Scanning Electron Microscopy (SEM)

**Figure 5:**
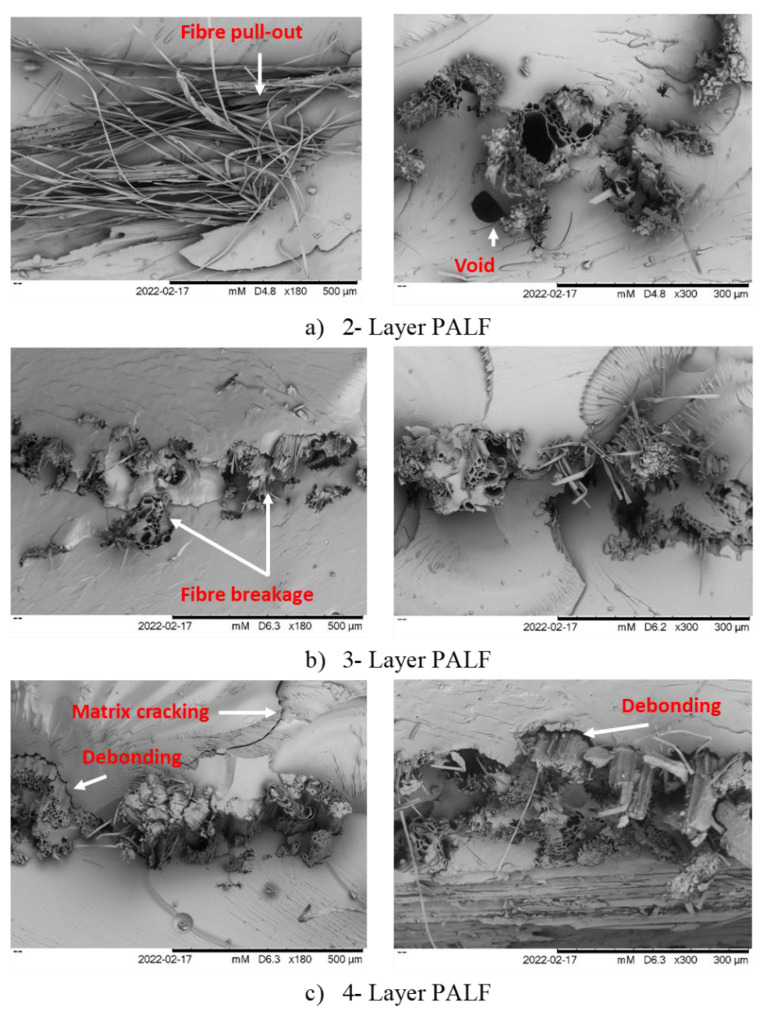
Tensile fracture of woven PALF/epoxy composites; (**a**) 2-layer, (**b**) 3-layer, (**c**) 4-layer.

Figure 5 shows the results of the tensile fracture surfaces for woven PALF-reinforced epoxy composites under scanning electron microcopy with a magnification of ×180, ×300 and a 15 kV. It was discovered that fibre pull-out, voids, debonding, and matrix cracking are essential factors influencing the interfacial adhesion between the matrix and fibre [11]. Moreover, fibre and matrix internal bonding was found to be very important in the NFRPC properties [7] because of its significance in the stress transfer from the matrix to the fibre, which ensures the most effective application of woven PALF-reinforced epoxy composite.

Fibre pull-out is one of the essential aspects influencing the failure mechanisms in NFRPC under the tensile test [2]. It was observed that there was no air bubble effect on the composites with 3L weft fibre orientation as indicated in Figure 4, but the micrograph of 3L warp showed that it has less fibre pull-out which caused a good interfacial bond between woven PALF and epoxy resin, thereby leading to a higher tensile strength of 33.76 MPa compared to other layering number and fibre orientations. Moreover, the 3L warp also showed a closer bonding between the fibre and matrix. Kumar et al., (2016) studied coconut sheath/banana fibre composites based on the layering patterns and found that the brittle failure was increased by the tightened bonded fibre due to the rich matrix regions [31]. The micrograph of the 2-layering woven PALF showed that the tensile strength for the warp direction was 23.97 MPa and weft was 20.98 MPa, which indicated an adhesion between the PALF and epoxy matrix, thereby leading to the production of a minimum value of tensile strength compared to another specimen.

The low tensile properties of the fabricated composites were also due to the voids and the fibre structure of the woven PALF as reported by Singh et al., (2020) in a study on the effect of the layering number on woven kenaf/jute fibre composites. It was discovered that the formation of voids and damage to the matrix in the woven PALF composite were due to the non-uniform distribution of fibre in the matrix [32]. Maliah et al., (2013) also showed that good dispersion and distribution of fibres are very important to have good mechanical properties [13]. The information in Figure 4 indicates the presence of an obvious gap between the woven PALF and epoxy matrix, which caused the lower value associated with the tensile properties and poor bonding interaction between the fibre and matrix, which subsequently had the ability to reduce the strength of the composites. It is also important to note that air bubbles were spotted in several areas in the resin region.

The pineapple leaf fibre (PALF) used to reinforce polymer composites is the most widely used type of fibre in the textile industry. This is because of its widespread availability, lower cost, good thermal and acoustic insulation, excellent tensile strength, and high toughness. PALF is also one of the most environmentally friendly types of fibre [33]. There are a variety of approaches to utilising PALF as a material for environmentally responsible development, and PALF is currently being utilised in a wide range of industries, such as the production of textiles, sporting goods, luggage, automobiles, cabinets, and mats, to name a few [34].

## 4. Conclusions

This study examined the influence of layering number and fibre orientation on the mechanical properties of pineapple leaf fibre reinforced epoxy composites. The 3L woven PALF has the highest tensile and flexural properties compared to other layerings. Due to the fibre and matrix compatibility, 3L woven PALF has a tensile strength of 34.40 MPa and a flexural strength of 79.25 MPa. The maximum layering content of woven PALF at 4L has the lowest tensile and flexural properties due to the inability of the composite materials to retain the reinforcement of the polymer matrix beyond the critical fibre composition, which results in minimal stress transfer between the fibre and matrix.

## Figures and Tables

**Table 1:** Composition of woven PALF-reinforced epoxy composite.

No of Woven Layering	Weight Fraction of Woven PALF (g)	Weight Fraction of Epoxy Resin (g)	Weight Fraction of Hardener (g)	Percentage of Woven PALF (wt.%)
2-layer (2L)	90	270	90	20
3-layer (3L)	120	270	90	25
4-layer (4L)	150	270	90	29.4

**Table 2:** Results of previous studies on the tensile properties of fibre-reinforced epoxy composites at different layering numbers.

Fibre Type	Layering Number	Tensile Strength (MPa)	Tensile Modulus (GPa)	Reference
Kenaf	Pure layer	30.00	1.5	[9]
Kevlar	Pure layer	250.00	6.5	
Oil Palm	Pure layer	22.61	2.3	[19]
Jute	Pure layer	53.31	4.2	
Ramie	5-layer	54.88	9.13	[20]
Sugar Palm	5-layer	39.42	9.75	
Flax	Pure layer	46.21	1.58	[21]
Jute	Pure layer	43.32	1.64	
Hemp	Pure layer	36.48	1.43	
PALF	4-layer	47.07	2.98	[22]
PALF	6-layer	124.72	1.36	[6]
Flax	6-layer	219.32	5.23	
Jute	5-layer	52.00	8.9	[11]
Ramie	5-layer	62.00	9.8	
PALF	2-layer	27.30	1.01	Current study
	3-layer	34.40	1.14	
	4-layer	28.90	0.93	

**Table 3:** Results of previous studies on the flexural properties of fibre-reinforced epoxy composites at different layering numbers.

Fibre Type	Layering Number	Flexural Strength (MPa)	Flexural Modulus (GPa)	Reference
Kenaf	Pure layer	20.00	1.0	[9]
Kevlar	Pure layer	110.00	7.5	
Oil Palm	Pure layer	43.00	2.5	[19]
Jute	Pure layer	78.00	5.1	
Ramie	5-layer	99.78	5.92	[20]
Sugar Palm	5-layer	78.88	4.40	
Flax	Pure layer	80.00	0.9	[21]
Jute	Pure layer	60.00	1.42	
Hemp	Pure layer	85.59	1.78	
PALF	4-layer	78.04	2.95	[22]
PALF	6-layer	52.25	2.51	[6]
Flax	6-layer	132.44	12.97	
Jute	5-layer	88.00	5.0	[11]
Ramie	5-layer	100.00	5.5	
PALF	2-layer	62.30	3.60	Current study
	3-layer	79.25	3.80	
	4-layer	59.00	3.40	

**Table 4:** Comparison of mechanical properties of PALF-reinforced epoxy composite with different parameters.

Parameter	Concentration of PALF	Observation	Reference
Unidirectional PALF	5–25 wt.%	− A 20 wt.% of PALF achieved highest tensile modulus for untreated and treated PALF (3090 MPa and 3440 MPa) and also for flexural modulus. − Impact strength decreased with the addition of PALF	[28]
PALF fibre with 2-5 mm in length	0–25 wt.%	− 10% PALF loading delivered better mechanical properties	[29]
PALF fibre with 35 mm in length	10–30 vol.%	− Average TS and FS found increasing with increasing fibre volume fraction	[30]
Woven PALF	2, 3, and 4 layer	− A woven 3-layer PALF with a 25 wt.% has the highest TS when compared to other concentrations	This study

## Data Availability

Data are contained within the article.
